# Small Molecules, Non-Covalent Interactions, and Confinement

**DOI:** 10.3390/molecules25143311

**Published:** 2020-07-21

**Authors:** Gerd Buntkowsky, Michael Vogel

**Affiliations:** 1 Institut für Physikalische Chemie, Technische Universität Darmstadt, 64287 Darmstadt, Germany; 2 Institut für Festkörperphysik, Technische Universität Darmstadt, 64295 Darmstadt, Germany

**Keywords:** confinement, solid-state NMR, molecular dynamics, interfaces and surfaces

## Abstract

This review gives an overview of current trends in the investigation of small guest molecules, confined in neat and functionalized mesoporous silica materials by a combination of solid-state NMR and relaxometry with other physico-chemical techniques. The reported guest molecules are water, small alcohols, and carbonic acids, small aromatic and heteroaromatic molecules, ionic liquids, and surfactants. They are taken as characteristic role-models, which are representatives for the typical classes of organic molecules. It is shown that this combination delivers unique insights into the structure, arrangement, dynamics, guest-host interactions, and the binding sites in these confined systems, and is probably the most powerful analytical technique to probe these systems.

## 1. Introduction

Porous silicates and alumosilicates include such diverse materials as the well-known microporous zeolites, a group of crystalline alumosilicates present in daily life, over mesoporous silica materials, such as the original ordered periodical mesoporous silica (PMS) [1,2] to controlled porous glasses and aerogels. They span a diameter range from fractions of a nanometer to ca. 50 nm and above. Owing to this large dispersion of well-defined diameters these systems are among the most versatile solid host-systems for studies of molecules in confinement.

In the present review an overview of confinement studies in PMS materials with a focus on neat and surface modified MCM-41 (Mobil Composition of Matter No. 41) [3] and SBA-15 (Santa Barbara Amorphous) [4,5] as hosts is given. Both MCM-41 and SBA-15 are characterized by well-ordered hexagonal pore-arrays, however, with different pore diameter distribution. Owing to their ordered structures, high porosity, high intrinsic surface area, low density, thermal stability, tunable pore sizes, and functional surface groups, PMS were successfully employed in a large variety of applications ranging from gas-storage and separation over heterogeneous catalysis to drug delivery (see, e.g., [6,7,8,9,10,11,12,13,14,15,16,17,18,19,20,21,22]), and many more.

PMS-type materials are ideal host systems for confinement studies since they combine narrow pore-diameter distributions and large specific surface with good chemical stability, easy handling, and chemical functionalization. They were employed in studies on the structural and dynamic properties of many different confined molecules, including water, alcohols, carbonic acids, protein solutions, and on thermophysical processes, such as freezing and melting points, glass transitions [23,24,25,26,27,28,29], or an electrochemical study of local pKa in confinement [30].

Of particular importance here are the periodic mesoporous silica materials MCM-41 [3] and SBA-15 [4] and their many derivates, which exhibit well-defined hexagonally arranged mesopore structures and three dimensional sponge like structures in porous glasses such as Vycor [31] or CPG-10–75 [32,33]. These materials opened up new research fields, as they allowed the introduction of larger molecular entities into well-defined pores. The prosperity of this field can be seen from the fact that, currently (March 2020), there are nearly 10,000 references in the Web of Science which have MCM-41 or SBA-15 in their title.

A large part of the interest in these materials stems from the fact that these materials are chemically very stable, because of the strength of the covalent Si-O-Si bonds, and that their surface silanol (Si-OH) groups are very potent reactive groups for chemical modification or functionalization of their surfaces. Thus, it is relatively easy to introduce the necessary functional groups by linker molecules, which serve, e.g., as possible binding sites for the chemical function of interest, such as a catalytic center. Such linker molecules can change the polarity or hydrophilicity of the surface, modify the hydrogen bonding properties of the surface, or add chemical functions, e.g., amide or carboxy functions [11] by binding functional groups such as amino, amide, carboxyl, phosphate, chloride, or peptide functions by post-synthetic grafting. Alternatively, it is also possible to add some of these functions directly during synthesis by co-condensation with appropriate additives [12,34].

A salient prerequisite for all these applications is a deep understanding of how the pores and pore surfaces interact with the confined molecules, which are, e.g., substrates of a catalytic reaction, a drug to be delivered or a fluid mixture which should be separated into its components.

This understanding is only obtainable by a combination of various complementary spectroscopic, thermodynamic, computational, and general physico-chemical characterization techniques, including multi-nuclear variable-temperature solid-state NMR (SSNMR), differential scanning calorimetry (DSC), powder X-ray diffraction (PXRD), small angle scattering (SAXS and SANS), thermogravimetric analysis (TGA) [35], and molecular dynamics (MD) simulations, as is shown by a number of recent papers (see, e.g., [36,37,38,39,40,41,42,43,44,45,46,47]). While X-ray diffraction techniques like XRD or SAXS reveal the ordered structures of these materials [48,49,50], nitrogen adsorption is employed to study the specific surface areas and pore diameters [51,52], DSC or TGA are used for the investigation of phase transitions inside the pores, NMR provides insights into the local ordering and dynamics on the molecular level, and computation interprets these results [35,53].

The purpose of this short review is to collect and report recent results on the investigation of guest molecules confined in mesoporous host systems with a particular emphasis on examples, where NMR techniques are prominently employed. As the description and theory of the NMR experiments employed for these investigations and their physical background are found in the literature, they are not described here in detail. Instead the reader is directed to a number of recent reviews on these experiments and references therein [54,55,56,57]. The same is true for the background of melting and glass transitions in confinement, where the reader is referred to papers [27,41,58,59] and references cited therein. The main advantage in the application of SSNMR techniques, which makes them complementary to diffraction techniques, is the fact that SSNMR works well on disordered systems or materials strongly affected by local impurities or multi-phase materials, on the one hand [39], and that it is able to analyze not only structural but also dynamical processes and in particular phase transitions on the other [35,53]. The drawback of NMR, in general, and SSNMR techniques, in particular, is their low sensitivity. For this reason microporous materials like zeolites [54,60,61,62,63] or mesoporous materials like MCM-41 and SBA-15 derivatives with high specific surfaces are commonly employed as host materials for NMR-confinement studies [64,65,66,67]. To battle this drawback, indirect detection methods under MAS, where the X-nucleus of interest is detected via the far more sensitive protons, a technique originally developed by Ishii and Tycko [68], were successfully applied to porous systems by the Pruski group to achieve remarkably sensitivity enhancements [69,70,71,72].

A recent alternative to this pure SSNMR technique for sensitivity enhancement is the application of hyperpolarization techniques like Dynamic Nuclear Polarization (DNP) enhanced SSNMR [73,74,75,76], which boost the sensitivity of SSNMR by several orders of magnitude [77,78,79,80,81,82,83,84], and in particular its variant SENS (Surface Enhanced NMR Spectroscopy) [85,86,87,88,89,90,91,92,93,94,95], or parahydrogen-induced polarization [96,97,98] (PHIP), whose potential for surface studies was demonstrated by Hunger et al. [99,100] and Stepanov and coworkers [101], or spin-exchange optical pumping (SEOP) [102,103].

In particular for functionalized systems, SSNMR techniques provided unprecedented details about the interaction of the linker molecules and the surface and its wetness [104,105,106]. Motokura et al. [107] employed ^13^C CP MAS NMR to investigate the catalytic transformation of epoxides under CO_2_ atmosphere on silica-supported aminopyridinium halides. Gath et al. [108] ascertained the properties of silylated amorphous silica materials. Wang et al. [109] studied a series of different linker molecules tethered on MCM-41 or SBA-15 by ^2^H MAS. Kandel et al. [110,111] investigated inhibitory processes in aldol reactions employing amine-functionalized silica supports. Jayanthi et al. [112,113,114] combined ^2^H and ^29^Si MAS NMR with MD simulations to study the dependence of the linker-surface interaction on the water concentration and on the temperature for *N*-(2-(triethoxysilyl)propyl)acetamide-d_3_ grafted onto MCM-41 and *N*-(2-aminoethyl-d_4_)propanamide grafted onto SBA-15. The Bluemel group pioneered in a series of seminal papers the application of CPMAS, in general and in particular, of HRMAS (high-resolution magic-angle spinning [115]), a solid-state NMR experiment, which employs the partial motional averaging of anisotropies for the investigation of physisorbed or chemisorbed molecules on surfaces, the characterization of novel porous catalysts [104,105,116,117,118,119,120,121,122,123,124,125]. Important contributions by the Coperet group were studies of various supported organometallic catalysts by SSNMR (see [86,87,90,92,126,127,128,129,130,131,132]) and by the Scott group [133,134,135,136,137,138], who developed a series of novel porous catalytic materials and investigated in detail the factors determining their adsorption and reactivity properties and by the Pruski [69,70,71,72,139,140,141,142,143,144,145] and Buntkowsky [146,147,148,149,150,151,152] groups, who employed conventional and DNP-enhanced SSNMR for the characterization of immobilized molecules. Another important aspect is that these materials are potential carriers for bioactive molecules, such as amino acids, peptides, or drugs [19,153,154,155]. Klimavicius et al. investigated silica confined ionic liquids by CPMAS NMR [156].

While the focus of this review is devoted to results obtained in the DFG special research unit FOR1583, there are also short reports about important contributions from outside this consortium. The rest of this review is organized as follows: Section 2 gives an introduction into the preparation and surface modification of the mesoporous host materials. Section 3 discusses the behavior of simple systems and Section 4 the behavior of complex systems, such as binary liquids or crowded solutions inside the confinement. The review is finished by a summary and an outlook into possible future developments of the field.

## 2. Porous Host Materials

### 2.1. Microporous Materials as Hosts

Owing to their immense importance both in daily life and in technology, zeolites are most probably the best characterized class of porous materials. They are well-ordered framework silicates with the composition (A^+^, E^2+^_0.5_)_x_ (AlO_2_)_x_ (SiO_2_)_y_ ∙ (H_2_O)_z_ (A^+^ = Na^+^, K^+^ and E^2+^ = Mg^2+^, Ca^2+^) and belong to the family of tectosilicates. A well-known example, found in nature, is faujasite (Na_2_Ca[Al_4_SiO_10_O_28_] ∙ 20 H_2_O) [157]. Zeolites find applications e.g., as molecular sieves [158,159], in heterogeneous catalysis [160,161], for gas storage [162], and as ion exchange resins [157]. An extensive recent overview about this fascinating class of materials is given in the recent special issue on zeolite chemistry

Owing to their narrow pore diameters and importance as catalysts for organic chemistry, most confinement studies employing zeolites use small molecules [163,164]. Typical recent examples are the deuterium NMR studies by Nishchenko et al. [165] and Lalowicz et al. [166,167] who analyzed the dynamics of tert.-butyl alcohol-d_9_ and methanol-d_4_ inside zeolites, respectively. Moreover, NMR field-gradient approaches yielded valuable insights into the diffusion of various small molecules in zeolites, including information about diffusion anisotropy, transport resistance at crystal surfaces, and pore connectivities [168,169,170,171,172,173,174].

### 2.2. Mesoporous Silica Materials as Hosts

While microporous zeolites are clearly still the technically most important class of porous host materials, their applications are limited to fairly small molecular sizes. For this reason the development of new classes of materials [3,6,7,8,9,10,16,17,18,19,20,21,106] with larger and adjustable pore sizes, such as mesoporous silica and mesoporous carbon materials, gained in importance. Depending on the material, they are characterized by adjustable pore sizes between ca. 10 and 1000 Å and, thus, close the gap between the microporous and the macroporous regime. Their combination of large specific volume and specific surface areas with high thermal stability and low specific weight creates a large application potential in physics, chemistry, pharmacy, polymer science, and related fields. Characteristic examples include applications in gas storage, in catalysis, in separation techniques, as additives to rubbers for tires media, and many more [11,12,13,14,15]. In confinement studies, SBA-15-type materials have the advantage of larger pore diameters, but MCM-41-type materials are better suited as models with narrow confinement. Additional merits of the latter are their generally better surface homogeneity and smoother inner surfaces.

### 2.3. Preparation and Chemical Functionalization of Mesoporous Silica; NMR Characterization

**Figure 1:**
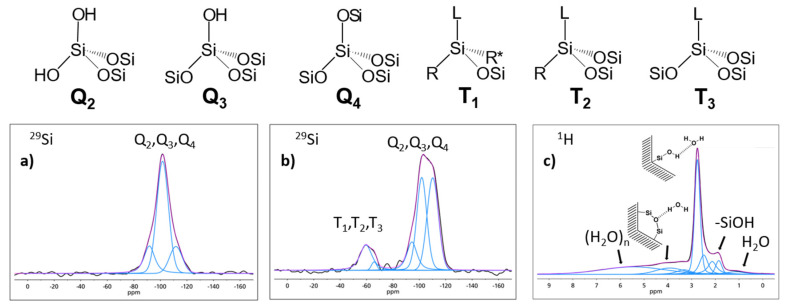
Upper panel: Different types of silica sites. Lower panel: ^29^Si CPMAS (10 kHz) spectra of (**a**) neat and (**b**) functionalized silica, showing the appearance of T_n_-groups by the functionalization. (adapted from Weigler et al. [181]). (**c**): ^1^H SSNMR spectrum of a non-dried MCM-41 at 10 kHz (black). Deconvolution (blue), sum of deconvolution (magenta) and assignment of water species to the peaks [36].

As mentioned above these mesoporous silica materials permit the comparatively simple synthesis of surface functionalized host systems with well-defined tunable narrow pore diameters [175,176,177,178], employing a synthesis protocol, which is based on Grünberg et al. [106] and Grün et al. [179]. Details of the synthesis and characterization are given in [176,180] and will not be repeated here. The changes of porosity, specific surfaces and the modification of the surface sites of the material can be monitored by the combination of nitrogen adsorption (BET and BJH) analysis and ^29^Si SSNMR spectroscopy. A typical example of such a synthesis is shown below (Figure 1), which displays the APTES ((3-aminopropyl)triethoxysilan) functionalization of MCM-41 materials. For this sample the BET measurements revealed a specific pore volume of 0.77 cm^3^/g, a specific surface area of 1000 m^2^/g and a specific pore diameter of 3.6 nm. From the ^29^Si SSNMR spectra the changes of the silanol groups during functionalization are determined by the change from Q-groups to T-groups.

At this point it is important to note that the freshly prepared samples in general contain a substantial amount of surface bound water molecules [36,39,182]. Since the latter can strongly influence the outcome of confinement studies, it is in generally necessary to check the hydration state of the sample by ^1^H MAS NMR measurements (the lower right panel of Figure 1 shows a typical example) and to employ special drying protocols for the preparation of “water-free” silica samples (for details see Brodrecht et al. [183]).

Since naturally-occurring porous hybrid materials like the skeleton of diatoms are based on modified silica materials consisting of silica and sillafins (polyamines) [184,185,186,187], the functionalization of mesoporous silica with peptides and peptoides [44,47,113,175,176,177,178,188,189,190,191,192,193,194] creates controllable well-defined model systems for the in-vitro study of, e.g., biomineralization.

There are two different strategies for obtaining such peptide-functionalized silica materials, namely an activation of the silica by virtue of a linker group, followed by a grafting of the previously synthesized peptide as shown, e.g., in [47,112] or the direct synthesis of the desired peptide inside the pores employing a modification of the standard SPPS protocol [176,177,194].

**Figure 2:**
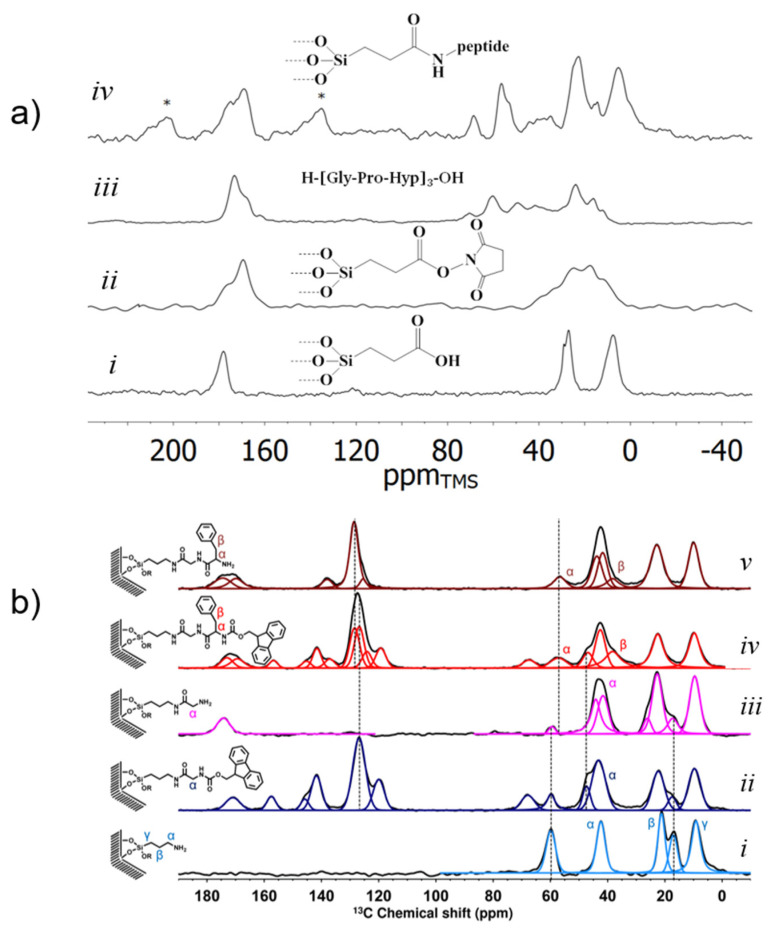
(**a**) ^13^C CPMAS NMR of the immobilization via the grafting approach of the nonapeptide h-[Gly-Pro-Hyp]_3_-oh on carboxyl functionalized mesoporous silica: (*i*) neat carboxyl functionalized silica support; (*ii*) TSTU (N,N,N′,N′-tetramethyl-*O*-(*N*-succinimidyl)uroniumtetrafluorborat) pre-activated silica; (*iii*) free nonapeptide h-[Gly-Pro-Hyp]_3_-oh; (*iv*) nonapeptide grafted on silica. Note: Signals marked with * refer to spinning sidebands. (adapted from [47]). (**b**) ^13^C CP MAS NMR of the steps of the in-pore SPPS (solid phase peptide synthesis) of functionalized SBA-15 (*i*), Fmoc-glycine functionalized species (*ii*), glycine functionalized species (*iii*), Fmoc-phenylalanine-glycine functionalized species (*iv*), phenylalanine-glycine functionalized species (*v*) (adapted from Brodrecht et al. [175]).

Figure 2 displays examples of both strategies and, in particular, how the success of the synthesis can be monitored by ^13^C CPMAS SSNMR spectroscopy. In the first example (Figure 2a), the collagen-model nonapeptide h-(Gly-Pro-Hyp)_3_-oh is grafted to silica [47]. The intensity reduction of the succinimidyl signal at ca. 15 ppm, which is visible by comparison of a.ii and a.iv, and the peaks for the nonapeptide in the carbonyl region and also in the aliphatic region indicate the successful immobilization of the peptide, which was proven by DNP enhanced natural abundance ^15^N spectra (not shown). The second example displays the steps of the SPPS inside mesoporous silica for the addition of one amino acid residue to an N-terminus (for details see Brodrecht et al. [175]).

**Figure 3:**
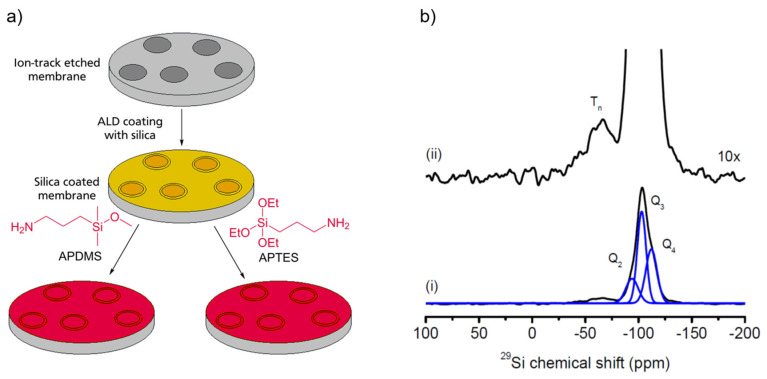
(**a**): Sketch of the three-step process to synthesize amine functionalized silica coated porous polycarbonate-membranes (see text for details). (**b**): DNP enhanced ^29^Si CPMAS spectra (i) revealing the characteristic T_n_-groups in the tenfold magnefication (ii) (adapted from [196])).

Although mesoporous silica materials offer a larger range of pore diameters they are still limited with respect to the accessible confinement sizes. In the case that functionalized pores with larger diameters are desired, they can be created in a hierarchical three-step process, which is sketched in Figure 3a. In the first step a membrane, such as a polycarbonate foil, is irradiated in a heavy ion accelerator, creating an ion-track. The carbonate material inside this iron track is then removed by etching, creating a channel through the polycarbonate foil. By selecting the ion dose, the irradiation directions, and the etching time, the number of channels, their dimensionality, and the channel diameters are selected [195]. In the second step these etched ion channels are coated with silica by atomic layer deposition (ALD). In the third step they can be functionalized by grafting of linkers, such as APDMS (3-aminopropyldimethyl silane) or APTES, to the silica inside the channels. Owing to their lower specific surfaces the detailed chemical characterization and monitoring of the surface functionalization by SSNMR is only feasible by means of DNP enhancement, which boosts the SSNMR sensitivity. As a typical example of these experiments, Figure 3b displays the DNP enhanced ^29^Si SSNMR spectra of the material. The broad low-field lines around ca. 60–75 ppm proves the formation of the characteristic T_n_-groups, which result from the binding of APTES to the silica inside the channels.

## 3. Simple Liquids in Confinement

The simplest case of a confined system is a single component liquid confined inside the pores of a host material, such as silica. In this case the behavior of the liquid is governed by the competition of the interactions between the liquid’s molecules and that of the host surface with the liquid’s molecules. In addition to the typical interactions between the liquid molecules, such as hydrogen bonding, hydroaffinity, polarity, and aromaticity, there are also steric effects, which influence the dynamics. In the remainder of this section, some characteristic examples of single component liquids, such as small polar, nonpolar, and aromatic molecules, confined in mesoporous silica are discussed. The competition of these interactions leads to pronounced changes of their phase behavior, in particular when confined in narrow pores, where a large percentage of the molecules is close to, or in contact with, the pore surface. The confinement in a pore causes in general a depression of the melting/freezing point of the confined molecules, respectively prevents melting at all if the pore diameter is too low, causing a glass-transition instead. As a consequence, many molecules, which are a solid in their bulk phase at a given temperature, become a liquid when confined inside pores.

The situation becomes even more complicated, when molecules are employed as solvents, e.g., of a chemical reagent such as a drug or in filtering processes. In this situation there will be a competition of solvent-surface and solute-surface interactions with solvent-solute and solvent-solvent interactions. In order to be able to understand these complicated systems, it is a prerequisite to understand the behavior and properties of confined simple liquids.

To obtain this understanding, various analytical methods such as DSC, TGA, near-infrared spectroscopy (n-IR), and variable-temperature XRD are combined with manifold NMR methods, including one- and two-dimensional spectroscopy, spin-lattice relaxometry, and correlation function analysis, as well as broadband dielectric spectroscopy (BDS).

### 3.1. Water inside Mesoporous Silica

Polar molecules such as water [36,40,197,198,199,200,201,202], alcohols like methanol [166,167], tert.-butyl alcohol [165] or octanol [203,204] and carboxylic acids, such as isobutyric acid [38,42], can form hydrogen bonds among themselves and also with the surface’s silanol groups (Si–OH).

Owing to its ubiquitous presence, its importance as a solvent and its importance for the life-sciences, water is the most interesting molecule for confinement studies. It is commonly used as a green solvent, employed both in technical processes and in medical applications. Moreover, due to its ability to build hydrogen-bonding networks with itself and also with surface-silanol groups and its rich phase-diagram, it is also a fascinating subject for basic scientific investigations.

Various aspects of water confined in mesoporous silica were investigated in a number of studies. An important outcome of these investigations was that the morphology of water inside the pores depends strongly on the pore diameter. For narrow pores a coexistence of two different water phases (a surface layer and nano-droplets or water-clusters) and for larger diameters a single water phase were detected by ^1^H MAS NMR [36,40] and ^2^H SSNMR [197,198]. It was feasible to assign different water species confined in mesoporous MCM-41 by virtue of combined ^1^H and ^2^H SSNMR experiments [199,200]. The exchange of the two spin species as a function of the hydration level was studied for MCM-41 filled with D_2_O [201] employing 2D selective soft-hard inversion recovery experiments [205,206] and the results were interpreted using a three site exchange model. In this model the highest exchange rate of 300 s^−1^ is found between single hydroxyl protons and water protons. Moreover, as they did not observe any coalescence for the lines corresponding to the surface-water chemical exchange rate, they could provide an upper boundary (<1000 s^−1^) for this rate.

When the confinement size is reduced, the melting temperature of water decreases, as described by the Gibbs–Thomson relation, until crystallization is fully suppressed [27,65,207]. Thus, severe geometrical restriction provides access to the properties of deeply cooled liquid water, which are of fundamental importance for an understanding of the anomalies of this liquid, but masked by rapid crystallization in the bulk [208]. In particular, it was proposed that the anomalies of water originate in a liquid-liquid critical point in the supercooled regime, which terminates a phase transition between high-density (HDL) and low-density (LDL) liquid forms [209]. Moreover, it was argued that the associated structural modifications have also a dynamical signature, explicitly, that there is a change in the temperature dependence of the structural α relaxation from a non-Arrhenius behavior characteristic for HDL to an Arrhenius behavior typical of LDL. While a number of studies reported such dynamical crossover of confined water, it remains a subject of controversial discussion whether the phenomenon is indicative of a HDL-LDL transition [210,211,212]. For example, alternative explanations based on confinement effects were given.

**Figure 4:**
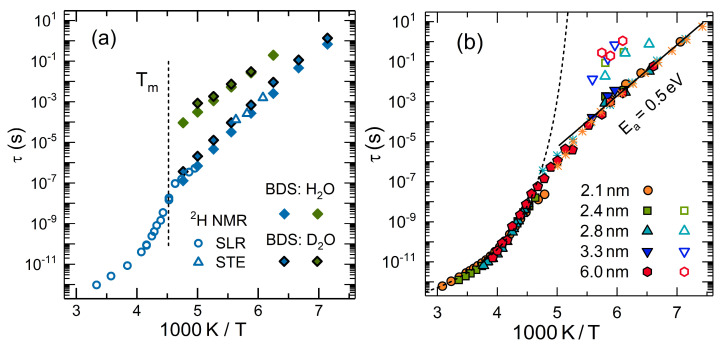
Correlation times of water reorientation in MCM-41 and SBA-15: (**a**) Results for H_2_O and D_2_O in MCM-41 pores with a diameter of 2.8 nm from BDS, ^2^H spin-lattice relaxation (SLR), and ^2^H stimulated-echo (STE) experiments [215]. The dashed line marks the melting temperature T_m_ of water in these confinements, as obtained from DSC (adapted from [215]). (**b**) Results for H_2_O and D_2_O in MCM-41 pores with the indicated diameters from ^2^H NMR [213] and BDS (stars) [212,215]. The dashed line is an interpolation of the high-temperature data with a Vogel-Fulcher-Tammann relation. The solid line is an Arrhenius fit of the low-temperature results, yielding an activation energy of 0.5 eV (adapted from [213]).

To tackle this problem, ^2^H NMR was used to investigate reorientation dynamics of unfreezable water (D_2_O) in MCM-41 and SBA-15 pores over wide temperature ranges towards the glass transition [45,176,181,183,213,214,215,216,217,218,219]. In particular, spin-lattice relaxation, line-shape analysis, and stimulated-echo experiments were combined to ensure broad dynamic ranges and the pore-size was systematically varied to study possible finite-size effects. Figure 4 shows temperature-dependent correlation times τ obtained from ^2^H NMR approaches to water reorientation in mesoporous silica. Clearly, there is a dynamic crossover near 220 K. Combining this NMR analyses with DSC and BDS studies [215], it turned out that, for a pore diameter of 2.8 nm, the dynamic crossover occurs near the melting temperature *T*_m_ so that liquid and crystalline water fractions with, respectively, faster and slower rotational dynamics coexist inside the pores below this temperature (see Figure 4a). It was concluded that partial crystallization causes the effect, explicitly, that the dynamics of water changes when ice forms and further restricts the accessible pore volume. To test this hypothesis, later work exploited that the melting temperature *T*_m_ can be altered when the pore diameter of the MCM-41 and SBA-15 material is varied [213]. It was found that the dynamic crossover occurs near 220 K, independent of the pore diameter (see Figure 4b). Hence, partial crystallization is not the reason of the change in the temperature dependence in the general case. One may be tempted to argue that the lacking pore-size dependence also excludes finite-size effects as possible origin. However, this argument does not hold because liquid water forms an interfacial layer, the thickness of which is largely independent of the pore diameter and, hence, the available space between the silica walls and the ice crystallites remains unaltered.

**Figure 5:**
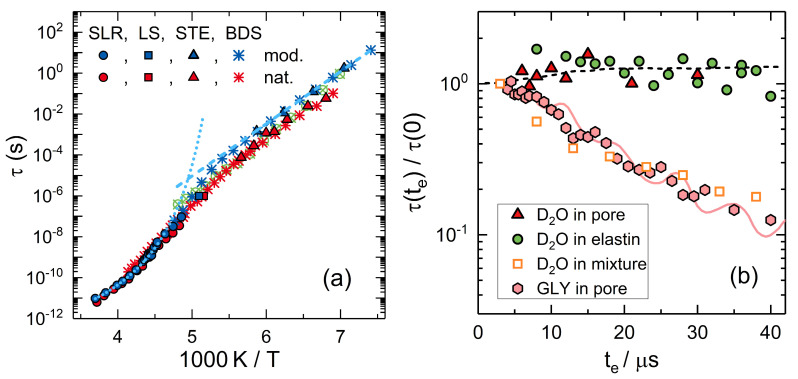
(**a**) Temperature-dependent correlation times of H_2_O and D_2_O reorientation in native MCM-41 (diameter 2.0 nm, red symbols) [214] and in APTES modified MCM-41 (diameter 2.2 nm, blue symbols) [181]. Results from ^2^H spin-lattice relaxation, line-shape analysis, and stimulated-echo experiments on D_2_O dynamics and from BDS on H_2_O dynamics in native MCM-41 [227] and D_2_O dynamics in modified MCM-41 [181] are shown. For comparison, data from combined NMR and BDS studies on water reorientation in an elastin matrix are included (green crossed circles) [222]. The dotted line is an interpolation of the high-temperature data with a Vogel-Fulcher-Tammann relation. The dashed line is an Arrhenius fit of the low-temperature results, yielding an activation energy of 0.5 eV. (**b**) Evolution-time dependent normalized correlation times τ(t_e_)/τ(t_e_→0) of D_2_O reorientation in an APTES modified MCM-41 (pore) [181], in an elastin matrix [220], and in a 2:1 molar D_2_O/DMSO mixture [228], and of glycerol (GLY) reorientation in MCM-41 (diameter 2.2 nm). The lines are expectations obtained from computer simulations [229]: (dashed) distorted tetrahedral jumps [220] and (solid) isotropic reorientation comprised of rotational jumps about angles of 2° (98%) and 30° (2%) [230].

To study the role of water-host interactions, analogous ^2^H NMR studies were performed for D_2_O in MCM-41 pores functionalized with APTES (see Section 2.3). It was found that water reorientation in native and functionalized MCM-41 pores is similar (see Figure 5a) [181]. In particular, the temperature-dependent correlation times τ show a crossover from non-Arrhenius behavior above ca. 220 K to an Arrhenius behavior below this temperature in both types of confinements. Moreover, a common activation energy of *E*_a_ ≅ 0.5 eV was observed in the low-temperature regime for water in MCM-41 pores with and without APTES functionalization, but also for water in many other confinements, e.g., at protein surfaces [220,221,222,223]. Therefore, the term ‘universal water relaxation’ was coined for the low-temperature process. However, there are ongoing vigorous discussions whether this dynamic process can still be identified with the structural α relaxation of water or to a secondary β relaxation, which has severe consequences for the value of the glass transition temperature *T*_g_ of confined water and, possibly, also bulk water (see below) [210,212].

To obtain information about the nature of the low-temperature dynamics of water, it was utilized that ^2^H stimulated-echo experiments provide access to not only the rates but also the mechanisms of molecular reorientation dynamics [224,225,226]. In particular, it can be exploited that the angular resolution of the experiment is determined by the length of the evolution time *t*_e_ in the stimulated-echo sequence.

It was reported that the observations for water in both silica and protein confinements are largely independent of the evolution time [45,181,214,219,220]. For example, the correlation times τ change in neither of these confinements when *t*_e_ is extended and, thus, the angular resolution is enhanced (see Figure 5b). This ineffectiveness of geometrical filtering indicated that water reorientation results from jumps about large angles of the order of the tetrahedral angle. Closer analysis implied that the universal low-temperature water reorientation can be described as distorted tetrahedral jumps or, similarly, as quasi-isotropic large-angle jumps [45,181,214,219,220]. However, it remains elusive whether or not the observed rotational motion is coupled to translational diffusion. The existence of such coupling is a prerequisite for the interpretation of the dynamic crossover in terms of altered structural α relaxation in response to a HDL-LDL transition. By contrast, an absence of such coupling implies interpretations based on a crossover from structural α relaxation to localized β relaxation. Moreover, this aspect has major consequences for the nature of the much-debated glass transition of water at *T*_g_ ≅ 136 K [231,232,233]. As the correlation times of the universal low-temperature water dynamics meet expectations for a glassy arrest at this temperature, a diffusive nature of this process entails a structural glass transition, whereas a localized nature implies an orientational glass transition, which is restricted to the rotational degrees of freedom.

### 3.2. Glycerol inside Mesoporous Silica

When investigating effects of geometrical restriction on liquid dynamics, it is desirable to compare behaviors of confined and bulk molecules over broad temperature ranges. In that respect, work on water has the drawback that its high tendency for crystallization hampers comparisons in the supercooled regime. On the other hand, use of good glass formers allows one to study molecular dynamics of both confined and bulk liquids in wide time windows. Major NMR contributions to this research field are discussed in a previous review article [67]. Therefore, we restrict ourselves to the case of the archetypal glass former glycerol in this contribution.

**Figure 6:**
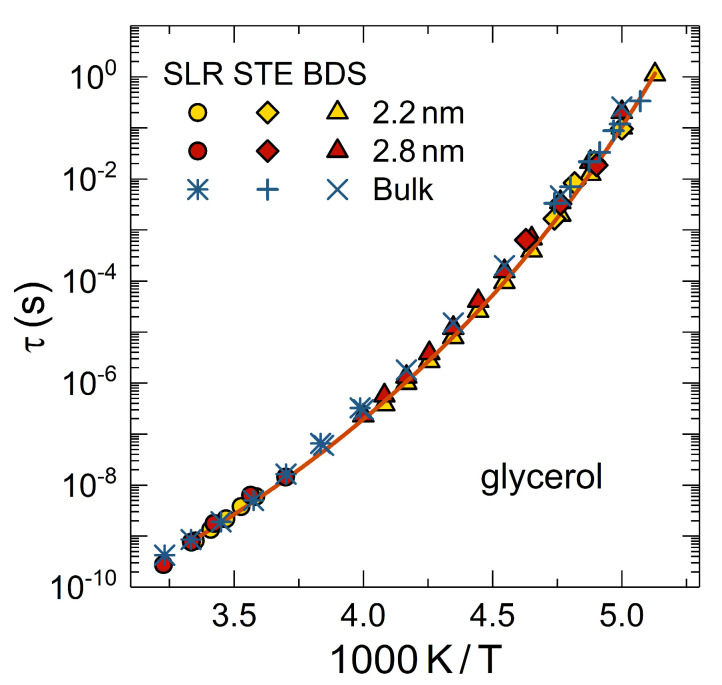
Temperature-dependent correlation times of glycerol-d_5_ in the bulk liquid and in MCM-41 pores with the indicated diameters. Results from ^2^H NMR (spin-lattice relaxation and stimulated-echo experiments) and BDS are compared. The solid line is an interpolation with a Vogel-Fulcher-Tammann relation.

Figure 6 shows ^2^H NMR correlation times of bulk and confined glycerol. It is evident that the time scale of glycerol reorientation is unaffected even in narrow MCM-41 pores with diameters of 2–3 nm and at low temperatures near the glass transition. The temperature dependence is well described by the Vogel-Fulcher-Tammann relation typical of molecular glass formers. To arrive at these results, it is, however, necessary to avoid contamination with water by careful drying of the precursor materials [183,217]. Possibly, the hygroscopic nature of mesoporous silica and the high sensitivity of glycerol dynamics to water admixtures offer an explanation for different conclusions relating to confinement effects on the glass transition of glycerol in BDS studies [234,235]. Moreover, ^2^H NMR stimulated-echo studies showed that MCM-41 confinement does not alter the mechanism for glycerol reorientation (see Figure 5b). Specifically, the evolution-time dependence of the correlation times τ(t_e_) of confined glycerol resembles that of bulk glycerol [230] but differs from that of confined water (see Section 3.1). The observed decrease of τ(t_e_) indicates that the reorientation process of glycerol is composed of consecutive small-angle jumps, e.g., the data for the bulk liquid were successfully described by an isotropic reorientation model, which assumes that 98% of the rotational jumps occur about an angle of 2° and only 2% of them involve an angle of 30° [230]. On the other hand, ^2^H NMR correlation functions were found to be more stretched for confined glycerol than for bulk glycerol [217]. Hence, confinement results in higher dynamical heterogeneity, which, most probably, reflects mobility gradients across the pores with slower dynamics at the pore walls than in the pore centers, as commonly observed in simulation studies on confined liquids [236]. Consistent with these results for silica confinement, it was reported that protein matrices leave the rate of glycerol reorientation unaltered but increase its heterogeneity [237,238].

### 3.3. Benzene, Biphenyl, and Naphthalene inside Mesoporous Silica

While polar molecules like water exhibit strong hydrogen bonding interactions with the confinement, aromatic molecules such as benzene [23,37], biphenyl [43] or naphthalene [239] only weakly interact with the surface due to their hydrophobicity and strong π-π-stacking interactions among themselves. Employing a combination of ^2^H SSNMR and DSC the phase behavior of benzene confined in mesoporous silica was studied [23,37]. These experiments revealed a drastic lowering of the transition temperatures of the rotor and translational phases of the confined benzene. In particular for narrow pores a glass-like benzene phase with a broad distribution of activation energies was elucidated [23]. In this glass-like phase the rotational degrees of freedom were strongly decoupled from the translational ones. The comparison of these results with investigations employing a larger-diameter host material revealed that these glass-like phases are formed by roughly three outer molecular layers and that inside a “normal” crystalline benzene phase is formed, which behaves like bulk benzene [37].

**Figure 7:**
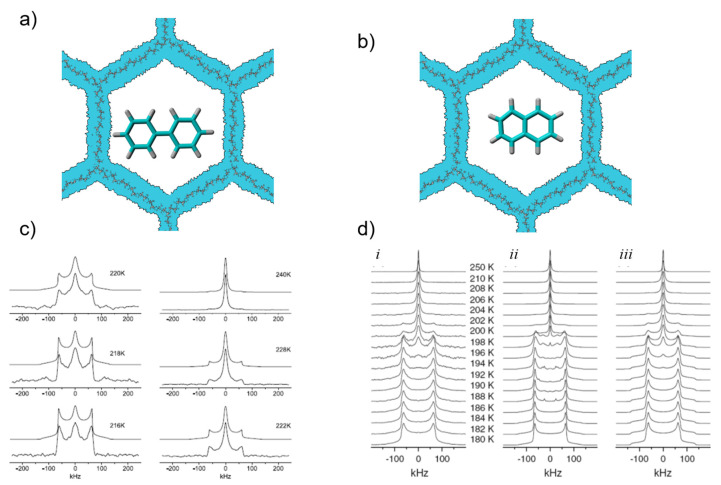
(**a**): cartoon of biphenyl [43] and (**b**) naphthalene [239] confined in MCM-41. In the case of biphenyl there are two possible rotational modes, namely, the rotation of a single phenyl ring and a molecular rotation. (**c**) experimental (lower traces) and simulated (upper traces) ^2^H solid-echo NMR spectra of the melting process of biphenyl-d_10_ inside silylated MCM-41 (d_aver_ = 29 Å) at different temperatures [43]. (**d**): experimental variable temperature ^2^H solid-echo NMR spectra of the melting process of naphthalene-d_8_ inside MCM-41 (d_aver_ = 33 Å) (i): experiment; (ii): simulated octahedral jump; (iii): two-phase model). All spectra are normalized to equal height (figure reproduced with permission from [176], copyright Walter de Gruyter and Company).

This interesting behavior prompted the study of the next larger homologues of benzene, namely of biphenyl confined in inside narrow (nominal 2.5 nm and 2.9 nm diameter) silylated and non-silylated MCM-41 pores [43] and of naphthalene confined inside narrow (nominal 3.3 nm pore diameter) MCM-41 pores [239]. With respect to confinement studies, a major difference between these two molecules is that biphenyl has an internal rotational degree of freedom, which naphthalene is lacking. The confinement of biphenyl caused a depression of the melting point by ca. 110–120 K from the bulk value of 342.6 K down to 222 K to 229 K (depending on the pore diameter). Moreover, a careful line-shape analysis of the ^2^H NMR solid-echo spectra measured just below the melting points elucidated indications for the presence of a pre-melting process in the form of isotropic motions of a fraction of the biphenyl molecules (Figure 7). The best simulation of the spectra was obtained by a two-phase model, with a broad distribution of rotational correlation times, resulting from a broad distribution of activation energies for the rotational motion. For the confined naphthalene an even stronger reduction of the melting point (152 K), compared to the bulk material was found. For the detailed line shape analysis of the ^2^H SSNMR spectra, two different models were employed, namely on the one hand a two-phase model with a broad distribution of activation energies, which is similar for benzene and biphenyl, and on the other hand a crystal-like jump model employing an octahedral jump geometry. Both models revealed a narrow melting point distribution of the confined naphthalene, indicating a relatively well ordered structure of the confined naphthalene molecules [239]. These results were interpreted such that the confined naphthalene molecules most probably form a plastically crystalline phase, similar to naphthalene in ball-milled silica [240,241,242,243]. The existence of these plastic phases of confined naphthalene were independently confirmed by a combination of DSC, Raman spectroscopy, and PXRD [244].

### 3.4. Pyridine inside Mesoporous Silica

While the nonpolar aromatic molecules described in the previous section interact only weakly with the silica surface, polar aromatic molecules such as carboxylic acids like benzoic acid [245], or nitrogen containing heterocycles such as pyridine [246,247,248], bipyridinyl [249], dimethylaminopyridine [250], or diethyl-2,6-di-tert.-butylaminopyridine [250], interact strongly with surface silanol groups by means of hydrogen bonds. In the case of the pyridine derivates, the ring nitrogen acts as a hydrogen bond acceptor. Since the ^15^N chemical shift is a very sensitive monitor of the hydrogen bond strength, these hydrogen bonds can be conveniently monitored by ^15^N CPMAS NMR [246,250].

In a series of seminal papers [246,247,248], Shenderovich and coworkers employed a combination of ^15^N- and ^29^Si-CPMAS and MAS NMR spectroscopy, line-shape analysis and structural modelling to describe the silica-surface morphology of different types of mesoporous silica on the atomic scale. While the ^29^Si MAS spectra revealed the ratio of Q_3_:Q_4_ groups and the pore wall ordering of the silica, the ^15^N NMR revealed the Brønsted basicity and surface morphology and surface defects of the silica. These investigations were paralleled by stray-field diffusion NMR studies, which investigated the diffusion tensor of pyridine as a function of the pore filling [251]. Later, Gurinov et al. [248] studied aluminum oxide containing SBA-15-type materials to investigate in more detail the effects of Lewis and Brønsted acidity by a combination of ^15^N and ^2^H NMR techniques. Lesnichin et al. [249] investigated the behavior of 2,2′-bipyridyl in confinement. They found that the molecule can only form one of the two possible hydrogen bonds to the surface and that the surface coverage grows strongly from one molecule per nm^2^ at room temperature to 1.6 molecules per nm^2^ at 130 K. Very recently, Shenderovich and Denisov studied the hydrogen bonding of pyridine in detail [252].

## 4. Complex Liquids in Confinement

In the previous section, the behavior of confined simple liquids was briefly reviewed. In the present section we now discuss the behavior of complex liquids, i.e., mixtures’ respectively liquid solutions of two or more liquid components [253]. The investigation of these mixtures is of particular interest as they are models for many natural or technical systems, e.g., oil-water mixtures. While the phase behavior of such complex liquids is often well understood in bulk phases, there is still a very large gap in knowledge for confined systems, where the competition of liquid/liquid versus liquid/pore surface interactions creates much more complex scenarios. Understanding the effect of the confinement on the complex liquid, and analyzing the structure, dynamics and spatial distribution of the solvents on the molecular level may help in developing new applications, e.g., in chemical industry, pharmacology or oil industry or might help in developing new strategies to deal, e.g., with crude oil spills. We start with model systems, namely confined water-alcohol and water-isobutyric acid mixtures to discuss the basic behavior of these confined systems. Then we discuss recent results on confined ionic liquids and their application as solvents in catalysis in the form of supported ionic liquid phases (SILPs) respectively supported ionic liquid catalysts (SILCs). Finally, we shortly summarize recent results on confined surfactants.

### 4.1. Confined Water-Octanol Mixtures

Water-octanol mixtures are important model systems for the investigation of the phase behavior of two immiscible liquids in confinement. For their quantification the water octanol partition coefficient or *p*-value K_ow_ is employed. (for details see the short review by Hermens et al. [254] and references therein). Hydrophobic liquids have a high K_ow_ and hydrophilic liquids have a low K_ow_. The K_ow_ values are employed, e.g., in pharmacology for estimating the distribution of drugs within the body. Drugs with high K_ow_ tend to accumulate in hydrophobic areas of the body such as lipid bilayers of cells and drugs with low K_ow_ tend to accumulate in hydrophilic areas with high water content, e.g., the blood serum. For a detailed discussion on the application of partition coefficients see Leo at al. [255].

**Figure 8:**
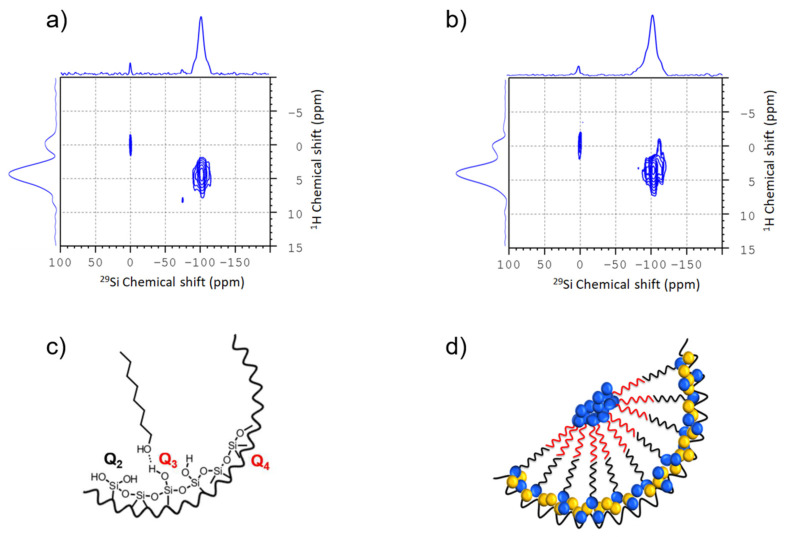
Upper panel: Room temperature ^1^H-^29^Si CPMAS FSLG-HETCOR experiment measured at 8 kHz spinning of dried SBA-15 filled with a mixture of 80:20 mol% of 1-octanol and water with a contact time of (**a**) 3 ms and (**b**) 9 ms. Lower panel: (**c**) schematic models for interactions of the pore surface of SBA-15 with 1-octanol. (**d**) Graphical visualization of a feasible bilayer formation of 1-octanol inside the pore. Water molecules are concentrated near the pore wall as well as in the pore center. The intermediated area between pore wall and pore center is occupied by the aliphatic hydrophobic chains of the 1-octanol molecules (adapted from Kumari et al. [203]).

Kumari et al. [203] studied the phase behavior of water/octanol mixtures confined in mesoporous SBA-15 by a combination of SSNMR and MD simulations (see Figure 8). By a combination of 1D SSNMR and FSLG-NMR [204] they could analyze the strength of the magnetic dipolar interactions between the different components and thus determine the distributions of the two liquids inside the confinement. The salient idea is to search for correlations between the chemically different types of ^1^H-nuclei (e.g., aliphatic protons of the alkyl chain or hydroxyl protons of the alcohol group or water) of the confined liquid and ^29^Si-sites on the surface of the material. These correlations are created by the magnetic dipolar interaction between these nuclei and are indicated as cross-peaks inside the 2D-NMR spectra. Thus, they are only visible when the corresponding nuclei are in the vicinity of each other. By varying the contact time different distances are probed. A detailed analysis of the 2D-spectra is beyond the scope of the current review and can be found in [203].

### 4.2. Water-Isobutyric Acid (IBA) Mixtures in Experiment and Simulation

Another example presents the study of binary mixtures of water and isobutyric acid (iBA, 2-methylpropanoic acid) by a combination of SSNMR spectroscopy and MD simulations. In bulk mixtures, this system has a well-known phase-diagram with a large miscibility gap as a function of temperature and mole fraction of the liquids. First NMR studies [38,39] of this system had indicated a micro-phase separation of the confined binary mixture with an anomalous temperature dependence of the self-diffusion coefficient and a bifurcation of the T_2_-relaxation upon a critical temperature of 42 °C, proposing a structural model in the form of concentric cylindrical liquid layers below the critical temperature inside the pores. The inner cylinder was tentatively assigned to the iBA rich and the outer cylinder hull to the water rich phase.

**Figure 9:**
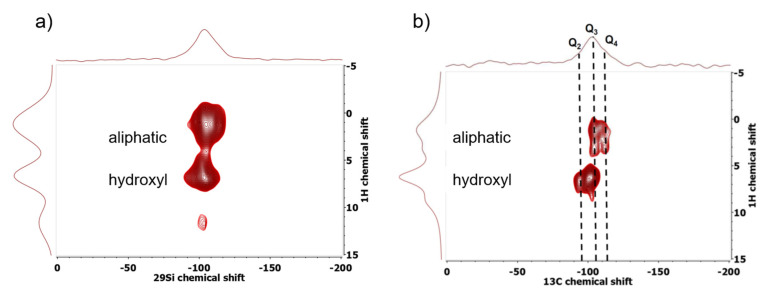
2D ^1^H-^29^Si FSLG HETCOR experiments of iBA/H_2_O mixtures confined in SBA-15 with contact times of (**a**) 3 ms (longer distances) and (**b**) 0.5 ms (shorter distances) clearly reveal that both hydroxy- and aliphatic protons are in contact with the surface silicon nuclei (56 wt % iBA, 9.4Tesla, 100 K, 8 kHz MAS, 89 kHz FSLG homonuclear decoupling [257]). (Figure adapted from [256]).

**Figure 10:**
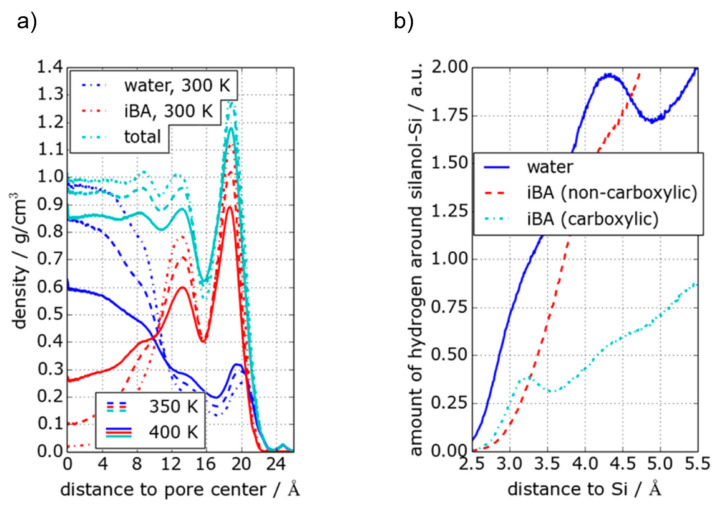
(**a**): density profiles for iBA and water calculated by molecular dynamics simulations. The center of Table 0. Å. (**b**): Density of hydrogen atoms as a function of the distance to the closest surface silanol group (figure reproduced from [256] with permission, copyright American Chemical Society).

This assignment was probed by Harrach et al. [256] by a combination of high-resolution SSNMR on frozen solutions (100 K to suppress any fluid mobility in the NMR experiments and obtain a momentary picture of the liquid distribution inside the pores) and MD simulations. By varying the contact time of ^1^H-^29^Si FSLG HETCOR (see Figure 9) they mapped out different distance regimes by virtue of the strength of the magnetic dipolar interactions between protons and the silica nuclei on the surface to reveal the molecular distribution inside the pores. The latter was interpreted by MD simulations (see Figure 10), which calculated the density profile of water and iBA as a function of the distance from the pore center. An example of these calculations is shown in Figure 10. They corroborate in principle the cylindrical model but reveal that the iBA rich phase and not the water rich phase is close to the pore wall. Furthermore, the calculations indicated that the iBA molecules orient preferential like an inverted brush-like structure, i.e., radially with the carboxylic group pointing towards the pore wall and the aliphatic chains pointing radially into the direction of the pore-center.

A detailed analysis of the entropic and enthalpic parts of the free energy revealed that this unexpected phase-behavior is mainly caused by the hydrogen-bonding enthalpy and is meliorated at higher temperatures where entropic terms become stronger, leading to a more thorough miscibility. For further details see the original paper by Harrach et al. [256]

### 4.3. Confined Water-Glycol Mixtures

Evidence for confinement-induced micro-phase separation and the confinement-enhanced tendency for crystallization were reported in ^2^H spin-lattice relaxation studies on mixtures of D_2_O with propylene glycol (PG), propylene glycol monomethyl ether (PGME), or dipropylene glycol monomethyl ether [217,258].

**Figure 11:**
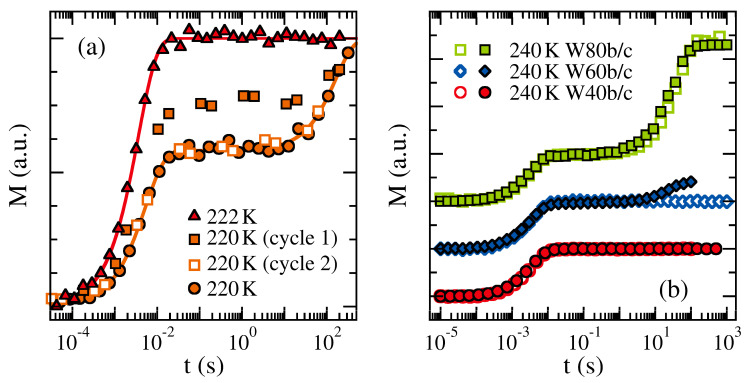
Buildup of ^2^H magnetization M(t) for water-glycol mixtures in the bulk and in MCM-41 pores (diameter 2.8 nm): (**a**) Confined PG-D_2_O mixture (45 wt % water) together with fits to monomodal (222 K) and bimodal (220 K) spin-lattice relaxation. At the lower temperature, the relative height of the fast and slow steps differs during the first and second cycles of a staggered-range measurement performed directly after temperature equilibration (squares), while this discrepancy does not occur during a later measurement. (**b**) Bulk and confined PGME-D_2_O mixtures at 240 K. The samples are denoted according to the weight percentages of water followed by the letters ‘b’ and ‘c’ for bulk (open symbols) and confined (solid symbols) mixtures, respectively.

For example, for a PG-D_2_O mixture with a water concentration of 45 wt %, ^2^H spin-lattice relaxation studies revealed that crystallization is fully suppressed in the bulk but occurs in pores at T < 220 K [217] (see Figure 11a). Specifically, bimodal ^2^H spin-lattice relaxation indicated that partial freezing results in coexisting liquid and crystalline fractions inside MCM-41 pores. Recording the buildup of the magnetization in a staggered way, it was even possible to follow the crystallization process on the basis of the observation that the slow step due to the crystalline fraction grows at the expense of the fast one associated with the liquid fraction in the course of time. Similarly, ^2^H spin-lattice relaxation results for PGME-D_2_O mixtures at 240 K indicated that freezing occurs in confinement but not in the bulk for a water concentration of 60 wt %, while such a difference was not observed at lower and higher water contents (see Figure 11b) [217]. Thus, at variance with the situation for pure liquids, confined aqueous glycol solutions with intermediate water concentrations show a higher proneness towards crystallization than their bulk counterparts. This effect was taken as evidence that, as a consequence of confinement-induced micro-phase segregation, the water concentration in some pore regions becomes sufficiently high to allow for ice formation.

### 4.4. Glass Transition of Confined Water-Alcohol Mixtures

^2^H NMR proved also useful to ascertain the glass transition of confined aqueous solutions [217,258]. In these studies, the strong slowdown of molecular dynamics related to the increasing viscosity can be monitored by a combination of, in particular, spin-lattice relaxation and stimulated-echo experiments. Moreover, depending on the deuteration scheme of the used compounds, it is possible either to observe the dynamical behavior of a particular component selectively or to probe that of both constituents at the same time.

**Figure 12:**
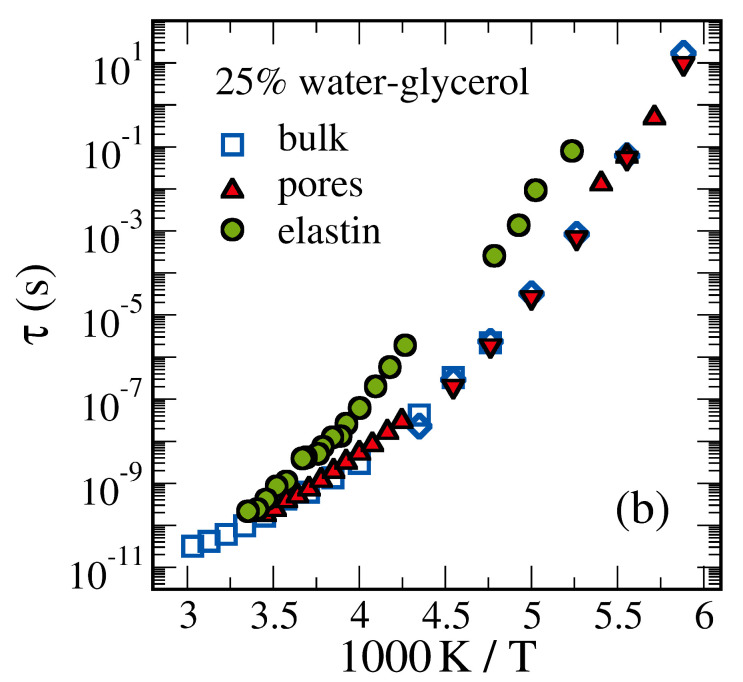
Temperature-dependent ^2^H NMR correlation times of 25 wt % water-glycerol-d_5_ mixture in the bulk liquid, in MCM-41 pores (diameter 2.8 nm), and in an elastin matrix (0.3 g_solvent_/g_elastin_) [217]. The ^2^H NMR data (squares, circles, and up triangles) selectively characterize the rotational motion of the deuterated glycerol compound. The BDS data (diamonds, down triangles) receive contributions from both water and glycerol reorientations.

Figure 12 compares ^2^H NMR correlation times of a water-glycerol mixture in the bulk with that in protein and silica confinements [217]. In all samples, 25 wt % of water were mixed with selectively labelled glycerol-d_5_. Hence, ^2^H NMR exclusively probes glycerol reorientation. The correlation times of the bulk mixture showed the characteristic non-Arrhenius temperature dependence of molecular glass-forming liquids over about 12 orders of magnitude. The agreement of the NMR data for glycerol dynamics with BDS results, which receive strong contributions from water reorientations, indicated coupled dynamics of the components. While glycerol reorientation was notably slowed down in an elastin matrix, the correlation times were unaltered when confining the liquid to MCM-41 pores [217]. This difference may suggest that glycerol interacts more strongly with elastin than with silica surfaces but it can also be caused by diverse confinement sizes in the studied samples. Specifically, the water-glycerol mixture forms only 1–2 solvation layers around the protein for the used concentration of 0.3 g_solvent_/g_elastin_, whereas interfacial and bulk behaviors can coexist in the MCM-41 pores with a diameter of 2.8 nm. Thus, the observed difference can result because slowed interfacial dynamics was probed for the elastin confinement, while bulk-like behavior in the pore center dominated the findings for the silica confinement. Consistent with the latter argument, spatially resolved analyses in molecular simulation studies of water-alcohol mixtures confined to silica pores revealed strongly retarded motion near the pore walls and bulk-like behavior in the pore center [259].

**Figure 13:**
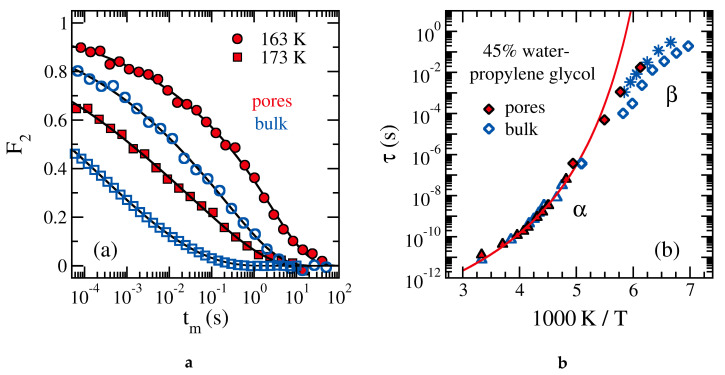
Results from ^2^H NMR studies on 45 wt % water-propylene glycol mixture in the bulk liquid and in MCM-41 pores (diameter 2.8 nm) [217]: (**a**) Rotational correlation functions F_2_(t_m_) from stimulated-echo experiments at ~163 K and ~173 K. The lines are fits to a Kohlrausch function. (**b**) Temperature-dependent correlation times from (triangles) spin-lattice relaxation, (diamonds) line-shape analysis, and (squares) stimulated-echo experiments. The line is a Vogel-Fulcher-Tammann fit of the spin-lattice relaxation results for the confined mixture, which probe its structural α relaxation. For comparison, results for the secondary β relaxation from BDS on the bulk mixture are included as stars [261] (adapted from [217]).

In ^2^H NMR studies on mixtures of alcohol molecules with heavy water, both components contribute to the observed signals because chemical exchange leads to perpetual redistribution of the provided deuterons [260]. Results obtained for a glass-forming mixture of water and propylene glycol in bulk and confinement are presented in Figure 13 [217]. While ^2^H spin-lattice relaxation does not yield evidence for confinement effects in the weakly supercooled regime, ^2^H rotational correlation functions from stimulated-echo experiments in the deeply supercooled regime decay slower for the mixture in silica pores than in the bulk liquid. Closer analysis showed that the common structural α relaxation of water and alcohol molecules is observed in the former temperature range, while faster water reorientation decouples as a secondary β relaxation from the viscous slowdown when approaching the glass transition temperature *T*_g_. Therefore, the authors concluded that the ^2^H stimulated-echo data do not yield evidence for a slowdown of the structural relaxation of the water-alcohol mixture in silica pores, but rather indicate changes of the secondary process [217].

### 4.5. Ionic Liquids and Surfactants in Confinement as Nonconventional Solvents

Ionic liquids (ILs) and surfactants such as poly(ethylene oxide) are versatile solvents in the field of green chemistry. Owing to favorable physical and chemical properties such as being environmentally benign and having a low vapor pressure, etc. (see, e.g., [262,263,264,265]) they are employed in a wide field of applications, ranging from basic preparative chemical synthesis to heterogeneous catalysis as a supported ionic liquid phase (SILP) catalyst [266]. In the latter application the IL is employed confined in an oxidic host material.

In the last decade, SSNMR has involved into one of the most versatile techniques to characterize the structure, dynamics, and phase behavior of ILs in general and SILP catalysts in particular, as demonstrated by the following characteristic examples.

Shylesh et al. [267] combined in situ FT-IR with ^31^P and ^29^Si MAS NMR to study the structure of sulfoxantphos (Rh-SX) in a silica supported ionic liquid film. Haumann et al. [266] studied the water-gas shift reaction employing SILP systems, consisting of the ruthenium catalyst ([Ru(CO)_3_Cl_2_]_2_) and [EMIM][NTf_2_] (1-ethyl-3-methylimidazolium bis(trifluoromethylsulfonyl)imide), respectively, [BMIM][NTf_2_] (1-butyl-3-methylimidazolium bis(trifluoromethylsulfonyl)imide) confined in a silica gel as a function of the pore loading. Le Bideau et al. [268] investigated the dynamics of silica confined ILs by a combination of variable temperature NMR spectroscopy and relaxometry. In these experiments they observed a strong depression of the freezing point of the IL, as compared to the bulk liquid and that the confinement causes only a small slowdown of its dynamics. Rosa Castillo et al. [269] studied the phase behavior of [BMIM][PF_6_] (1-butyl-3-methylimidazolium hexafluorophosphate) on silica and clay by multinuclear (^1^H, ^13^C, ^31^P) SSNMR spectrometry. Waechtler et al. [270] used a combination of DSC and variable temperature ^2^H and ^19^F solid-state NMR to compare the phase behavior of [C_2_Py][BTA]-d_10_ (*N*-ethylpyridinium-bis(trifluoromethanesulfonyl)amide) in the bulk and confined in mesoporous silica gel. While two phase transitions were found for the bulk IL, one at 288–289 K indicating the onset of an intermolecular rotation and one at 295 K indicating the melting of the IL, the confined IL exhibited only a single phase transition in the lower temperature range (215–245 K).

Very recently, Hoffmann et al. [271] investigated the behavior of nonionic surfactants doped with radicals confined in SBA-15 whose surface was modified with (APTES) by a combination of DSC, SSNMR and dynamic nuclear polarization (DNP) [85,87] enhanced SSNMR spectroscopy.

## 5. Summary and Outlook

This paper reviews recent advances to the characterization of small molecules confined in microporous and mesoporous materials employing solid-state NMR techniques. It is shown that there is an exciting interplay between guest/guest and guest/host interactions, which can drastically change the physico-chemical properties of the confined systems and that solid-state NMR spectroscopy and relaxometry, combined with other techniques, such as nitrogen adsorption, differential scanning calorimetry, dielectric spectroscopy, hyperpolarization, and others, are ideal analytical tools enabling the differentiation between bound, adsorbed and free molecules inside the pores, as well as the observation of diffusion processes inside the pores of mesoporous and microporous materials. A number of examples, mainly from the groups of the two authors, were given to highlight the application of these techniques. These examples were supplemented by short references to the work of other groups in the field in order to give a broader picture of the field of imprisoned molecules.

Finally, we will end our review with giving some thoughts, where the field is moving in the next few years. Here the dramatic technical advances in sensitivity enhancement of NMR spectroscopy will enable the investigation of even more complex systems, e.g., hierarchical confinements, where the mesoporous silica-material itself is confined inside larger pores of, e.g., a polymer or paper to form a smart membrane.
